# Apomixis: oh, what a tangled web we have!

**DOI:** 10.1007/s00425-023-04124-0

**Published:** 2023-03-31

**Authors:** Terzaroli Niccolò, Aaron W. Anderson, Albertini Emidio

**Affiliations:** 1 Dipartimento di Scienze Agrarie, Alimentari e Ambientali, Università degli Studi di Perugia, Borgo XX Giugno 74, 06121 Perugia, Italy; 2 Fulbright Scholar From Department of Plant Sciences, University of California, Davis, USA; 3 Consorzio Interuniversitario per le Biotecnologie (CIB), Trieste, Italy

**Keywords:** Environmental stresses, Epigenetics, Gene regulation, Hormones, Parthenogenesis, Reproduction

## Abstract

**Main conclusion:**

Apomixis is a complex evolutionary trait with many possible origins. Here we discuss various clues and causes, ultimately proposing a model harmonizing the three working hypotheses on the topic.

**Abstract:**

Asexual reproduction through seeds, i.e., apomixis, is the holy grail of plant biology. Its implementation in modern breeding could be a game-changer for agriculture. It has the potential to generate clonal crops and maintain valuable complex genotypes and their associated heterotic traits without inbreeding depression. The genetic basis and origins of apomixis are still unclear. There are three central hypothesis for the development of apomixis that could be: i) a deviation from the sexual developmental program caused by an asynchronous development, ii) environmentally triggered through epigenetic regulations (a polyphenism of sex), iii) relying on one or more genes/alleles. Because of the ever-increasing complexity of the topic, the path toward a detailed understanding of the mechanisms underlying apomixis remains unclear. Here, we discuss the most recent advances in the evolution perspective of this multifaceted trait. We incorporated our understanding of the effect of endogenous effectors, such as small RNAs, epigenetic regulation, hormonal pathways, protein turnover, and cell wall modification in response to an upside stress. This can be either endogenous (hybridization or polyploidization) or exogenous environmental stress, mainly due to oxidative stress and the corresponding ROS (Reacting Oxygen Species) effectors. Finally, we graphically represented this tangled web.

## Introduction

Reproduction is the goal of all living organisms, including plants. To ensure the propagation of the species, reproduction needs to be both reliable and flexible. The Eukaryotes’ most common reproductive strategy is sexuality, or amphimixis, which requires the fusion of male and female gametes. As a substitute, one in ten thousand species use asexuality to reproduce (Hojsgaard and Schartl 2021). Among the asexual modes of reproduction, apomixis, i.e., asexual reproduction through seeds, is the most intriguing and has high practical potential. Apomictic reproduction involves the development of an embryo from a cell with a somatic chromosome number, maintaining the maternal genetic setup. There are several ways to produce apomictic seeds. The most common, *adventitious embryony*, describes a situation where one or more embryos are formed directly within the nucellus or the integument, without forming a gametophyte. This kind of apomixis is not typically found in agronomically important crops, with the exception of several *Citrus* species and mango (Naumova 2017). Alternatively, if the maternal embryo originates from a diploid egg cell differentiated in an unreduced embryo sac, the process is defined as *gametophytic apomixis* (Nogler 1984; Bicknell and Koltunow 2004). There are two modes of gametophytic apomixis. The unreduced embryo sac can develop from a somatic nucellar cell that acquires the developmental program of a functional megaspore. This mechanism is also referred to as apospory. In apospory, sexually derived embryos and their asexual counterparts can coexist and compete for the endosperm resources (Hojsgaard and Hörandl 2019). Alternatively, if the embryo sac forms from a megaspore mother cell with suppressed or modified meiosis, the pathway is referred to as diplospory (Barcaccia and Albertini 2013).

Irrespective of how the embryo sac is formed, a parthenogenetic pathway is needed for the egg to develop into an embryo, and initiate seed development (Koltunow et al. 2011). After apomeiosis and parthenogenesis, successful seed production requires endosperm development. An endosperm can form without paternal contribution or, as in approximately 90% of apomictic species, through pseudogamous fertilization of the embryo sac central cell, with reduced or unreduced pollen (Bicknell and Koltunow 2004; León-Martínez and Vielle-Calzada 2019). In this case, only the endosperm is fertilized by the pollen and the embryo is a genetic copy of the mother with no male inheritance.

One of the major advantages of meiosis is genetic recombination, which can create new genotypes better adapted to novel conditions. Recombination also helps to avoid genetic degeneration through the accumulation of deleterious mutations (Hill-Robertson effect and Muller’s ratchet). Another frequently cited explanation for the advantage of sexual reproduction is the Red Queen hypothesis: parasites and pathogens attack the most common genotypes, so asexual individuals are more likely to be fragile and at a fitness disadvantage (Houliston et al. 2006). However, asexual species have developed several strategies to attenuate the consequences of avoiding meiosis, such as the “mutation-based” diversity and clonal competition (Hojsgaard and Schartl 2021).

In contrast with the Muller’s ratchet theory, mutation accumulation in apomictic plants -unlike in the other vegetative reproductions- seems not to occur, at least at high frequencies. This was demonstrated with the transcriptome of the facultative apomictic *Ranunculus auricomus* where Hodač et al. (2019) found no genome-wide mutation accumulation. In addition, most apomictic plants retain low-frequency instances of meiotic recombination (facultative apomixis) whose expression is influenced by environmental signals (Carman 1997; Mateo de Arias et al. 2020). This flexibility was found in *Hieracium pilosella* (Houliston et al. 2006), where a rate of sexual progenies of 0.2–6% is sufficient to respond to environmental changes. In addition to the other benefits, apomixis maintains seed dispersal, and, therefore, its advantage against pathogens infection, such as viruses (Kumar 2017).

From an evolutionary point of view, apomixis can be seen as a modification of the sexual developmental process (Koltunow and Grossniklaus 2003; Hand and Koltunow 2014; Brukhin 2017; Hojsgaard 2020). Identifying and characterizing specific recombinants has demonstrated that apomeiosis, parthenogenesis, and, more rarely, fertilization-independent endosperm formation can be uncoupled, suggesting that they are controlled by independent loci (Albertini et al. 2001; Koltunow et al. 2011; Conner et al. 2013; Ogawa et al. 2013). Recombination around apomixis loci is usually suppressed due to heterochromatic and highly repetitive genomic regions, resulting in high allelic divergence or hemizygosity throughout the genomic region (León-Martínez and Vielle-Calzada 2019). Examples include *Tripsacum* (Grimanelli et al. 1998), *Brachiaria* (Pessino et al. 1997), the apomictic controlling locus (ACL) of *Paspalum* (Calderini et al. 2006; Ortiz et al. 2017), or the apospory-specific genomic region (ASGR) of *Pennisetum squamulatum* (Akiyama et al. 2004) and *Cenchrus ciliaris* (Akiyama et al. 2005).

Recently, significant progress has been made to engineer apomixis, supporting the idea that the trait is genetically controlled by a few loci (“Genetic” hypothesis from hereafter). The effect of altered expression of specific genes support this hypothesis. These include *Pennisetum BABY BOOM* (BBM, Conner et al. 2015) that led to partial parthenogenesis in sexual plants and the *Arabidopsis DYAD/SWITCH1* (*SWI1*) that is a candidate for switching to apomeiosis (Ravi et al. 2008). Combining genes, such as in *MiMe* (*Mitosis instead of Meiosis*) mutant, resulted in *Arabidopsis* plants with apomeiotic behavior (d’Erfurth et al. 2009) that produced both diploid female and male gametes genetically identical to their parent. This approach was taken one step further in rice (*Oryza sativa*), where the *OsMiMe* triple mutant was crossed to a haploid inducer line expressing a modified version of the centromeric histone *CENH3* (Mieulet et al. 2016; Khanday et al. 2019; Xie et al. 2019). In this case, the haploid inducer genome is eliminated in the zygote, resulting in a clonal offspring and no doubling (Mieulet et al. 2016). Further, Xie et al. (2019) created the quadruple rice mutant *OsSPO11-1/OsREC8/OsOSD1/OsMATL,* renamed *Apomictic Offspring Producer* (*AOP*), with an enhanced efficiency in producing apomictic offspring. A similar approach was used by Khanday et al. (2019), who combined the *OsMiMe* mutant with the expression of BBM1 in the egg cell to achieve asexual propagation without genetic segregation. Although apomictic rice has been obtained by engineering genes mimicking aspects of apomixis, the obtained plants were not yet suitable for agronomic applications due to the low rate of clonal progenies over the sexually produced ones. This seems to be solved in the evolved system reported by Vernet et al. (2022), where as high as 95% of progenies were produced asexually by engineered rice plants. Moreover, the newly discovered *PARTHENOGENESIS* (*PAR*) gene of dandelion relies on a different molecular mechanism than BBM (Underwood et al. 2022), but could be just as useful in engineering apomixis *ex-novo*. Other candidate genes could be explored to engineer apomixis such as ORIGIN *RECOGNITION COMPLEX3*, which supports imbalanced endosperm development in aposporous *Paspalum simplex* (Siena et al. 2016) and *QUI GON JINN* that is necessary for the emergence of unreduced embryo sacs in *Paspalum notatum* (Mancini et al. 2018). Deletion of *LOSS OF APOMEIOSIS* (*LOA*) and *LOSS OF PARTHENOGENESIS* (*LOP*) loci of *Hieracium prealtum* results in the reversion of apomictic plants to sexual reproducing ones, which could support the hypothesis of superimposed apomixis over sexual reproduction (Koltunow et al. 2011).

In terms of evolution, the various forms of apomictic reproduction are found throughout angiosperms with no clear evolutionary pattern, suggesting multiple independent events (Hojsgaard et al. 2014). Several species, populations and individual plants show a blend of adventitious embryony, apospory, and diplospory (Hojsgaard et al. 2014), undermining the “genetic” hypothesis. Based on these observations, an alternative hypothesis, i.e., apomixis is ancestrally polyphenic with sex and it can be maintained by a different state of metabolic homeostasis (Mateo de Arias et al. 2020), was proposed (Carman 2007; Albertini et al. 2019). In fact, polyphenisms are traits that dramatically change in response to environmental signals (Projecto-Garcia et al. 2017). Therefore, a metabolite-driven upstream epigenetic process can act as the controlling switch between the sexual/apomictic reproduction involving enzyme function modifications and genome accessibility (“Polyphenic” hypothesis from hereafter, Albertini et al. 2019). Environmental factors indeed affect the penetrance of apomixis (Gounaris et al. 1991; Mateo de Arias et al. 2020): for example, in facultative apomicts, the proportions of meiotic derived megaspores, embryo sacs, and seeds produced by a clone can fluctuate, suggesting that genetic control is insufficient to explain the entire phenomenon (Hojsgaard and Hörandl 2015). A third hypothesis suggests that apomixis could be triggered by a deregulation (in time or space) of the sexual meiotic pathway replacing meiosis and syngamy with apomeiosis and parthenogenesis (from hereafter referred to as Asynchronous hypothesis, Hojsgaard 2020). These two latter hypotheses could appear quite different; however, they both lay their foundations on genomic shocks that lead to various level of epigenetic (de)regulation. On one hand, the Polyphenic hypothesis is endorsed by mutants affecting methylation patterns. On the other, the Asynchronous one is supported by transcriptional comparisons and ovule studies, such in aposporous (Galla et al. 2015, 2017; Schinkel et al. 2017) and diplosporous (Grimanelli et al. 2003; Bradley et al. 2007; Sharbel et al. 2009, 2010; Grimanelli 2012) species.

Transcriptome-wide changes at the onset of apomixis have been documented in several apomictic species (Hand and Koltunow 2014) and a global decrease in gene expression at the early stages of development in apomictic versus sexual individuals has been recorded in *Boechera divaricarpa* (Sharbel et al. 2010), *Hypericum perforatum* (Galla et al. 2017), *Eragrostis curvula* (Rodrigo et al. 2017; Garbus et al. 2019) and *Paspalum Rufum* (Soliman et al. 2021a, b). Transcriptional profiling of *Arabidopsis* embryos demonstrated that heterochrony of specific transcripts is more significant than spatial regulation (Spencer et al. 2007), suggesting that timing of gene expression is critical in early development. Additionally, the apomictic developmental progression is typically faster than the sexual one (Carman et al. 2011; Mateo de Arias et al. 2020). As a result, including developmental stage as a covariate has become the norm when comparing sexual and apomictic individuals or populations (Sharbel et al. 2009, 2010; Tang et al. 2017; Mateo de Arias et al. 2020; Podio et al. 2021). Polyploidization and hybridization are positively correlated with the origin of apomixis and can both be associated with extensive transcriptional reprogramming (Hojsgaard and Hörandl 2019; León-Martínez and Vielle-Calzada 2019). Both events can enhance heterozygosity. Therefore, DNA methylation and small RNA pathways might be useful to confer adaptive advantages to apomictic populations (Verhoeven and Preite 2014). For example, chromosomal doubling of a sexual diploid genotype of *Eragrostis curvula* led to an apomictic tetraploid plant. The authors suggested that this was due to novel epigenetic regulation associated with the transition to polyploidy (Zappacosta et al. 2014). Variations in methylation patterns in *Eragrostis curvula* were also detected after autopolyploidization by Cervigni et al. (2008), Ochogavía et al. (2011) and (Carballo et al. 2021). Similarly, methylation analyses revealed that new epialleles emerged after tetraploidization of a diploid *Paspalum notatum* (Rodriguez et al. 2012) and *P.* *rufum* (Siena et al. 2008). Several genes involved in aposporous development also seem to be regulated by ploidy (Laspina et al. 2008), and the dosage of genetic factors has been proposed to influence the penetrance of apomixis (Molins et al. 2014). In *Pennisetum squamulatum*, the apospory carrier chromosome alone was sufficient to establish an apomictic lineage, even if additional hybridization signals may have occurred (Goel et al. 2003).

In summary, the origin of apomixis remains enigmatic. Describing this transition in a recently evolved subset of eukaryotes could lead to ambiguous speculation due to the lack of proper understanding of the actual evolutionary path. Based on the de novo or Genetic theory, specific mutations to factors underlying the sexual pathways should lead to apomixis. In contrast, the polyphenetic and the asynchronous viewpoints suggest that sex and apomixis rely on the same genes and consequently, mutations in meiosis or syngamy genes may also be deleterious to apomixis. Until now, all evidence suggests the three hypotheses (genetic, polyphenic and asynchronous) can coexist and intersect themselves to explain apomixis (Hojsgaard 2020).

Is it possible that we are only looking at one side of a multifaceted problem such as apomixis? Could the true cause be combined effects of asynchronicities and environmental stresses interacting with small RNAs, epigenetic regulatory systems, hormone signaling, and other endogenous effectors? Here, we review recent findings on the role of all these mechanisms and the complex web of relationships among them in inducing apomictic behaviors.

### Endogenous stresses (hybridization/polyploidization)

While polyploidization is unsustainable for animals and species hybridization is rare, these phenomena are widespread among plants. Related species can combine to form a new hybrid plant that is initially sterile due to the imbalance of the genomes. This inability to pair with one another is due to differing ploidy, number of chromosomes, or the lack of homology between chromosomes. Rare doubled gametes can lead to triploid bridges and then to new fertile euploids (Hojsgaard 2018). The uncoupling of apomeiosis and parthenogenesis can boost this process in triploid individuals by enhancing the frequency of doubled gametes and permissive development of endosperm, thus producing more viable seed. After the settlement of the neo-poly-euploids, genome resilience can recover the “regular” sexual functionality, otherwise apomixis can then be retained (Hojsgaard 2018). Apomixis can be seen as a consequence of polyploidization/hybridization since it requires the subsequent re-coupling of apomeiosis, parthenogenesis and endosperm formation in neopolyploids. Also because polyploidization is boosted by the first genomic shock in triploids, though not all polyploids are apomictic (Hojsgaard 2018). On the contrary, hemizygosity and repressed recombination seem to be a consequence of apomixis with the main goal of avoiding segregation of the three components of apomixis and retaining all functionality and consequent fitness (Schmidt 2020).

Gametophytic apomixis is common in genera showing adventitious embryony (Asker and Jerling 1992) and in some rare cases, it can be sustained in diploid conditions. This condition is made possible by hybridizing two distant ecotypes or related species with different reproductive characters, or when the apomictic populations represent diploid-aneuploids, such as in the *Boechera* genus (Carman 1997; Kantama et al. 2007; Lovell et al. 2013). The additive asynchronous expression of the two parental genomes could lead to precocious embryo sac initiation and parthenogenesis (Carman 1997; Hojsgaard et al. 2014).

As stated by Meier et al. (2011) and confirmed by Zappacosta et al. (2014), in *Eragrostis curvula,* the stress due to chromosomal doubling, and subsequent in vitro culture, could explain the shift from sexual reproduction to almost 90% apomixis. The genotypes in question originated from a “back-and-forth” (4x-2x-4x) plant series. The initial tetraploid was apomictic; the following anther culture led to a sexual diploid that, after colchicine treatment led to new tetraploid individuals. After four years, the authors investigated genetic and epigenetic variations and found that a higher methylation rate occurred in the doubled genotype than in the control plants; moreover, they noticed that higher ploidy plants expressed more retrotransposons belonging mainly to Gypsy and Copia families (Zappacosta et al. 2014). Using the same genotypes, Rodrigo et al. (2017) registered an increase in sexual reproduction during tissue culture. This “shift” from apomictic to sexual reproduction could be biased by mutations or epigenetic changes induced by in vitro culture (Ong-Abdullah et al. 2015; Rodrigo et al. 2017).

Hybridization and polyploidization can hence be seen as a kind of endogenous stress. It can alter the ratio between apomictic/sexual embryo sacs in favor of the latter, and has been observed in facultative tetraploids cultivars of *Eragrostis curvula* (Rodrigo et al. 2017). Hybridization acts on the heterocromatinized and suppressed regions of the parents resulting in phenotypic instability (Comai et al. 2003). The epigenetic alterations in hybrids, together with a significant chromosome rearrangement, might be responsible for the deregulation in time and space of the sexual genetic program and lead to apomixis induction (Mau et al. 2021). The endogenous stresses are then the major representatives of the Asynchronous hypothesis.

### Exogenous stresses

In many studies (Cervigni et al. 2008; Schmidt et al. 2014; Galla et al. 2015, 2019; Tang et al. 2017; Ortiz et al. 2019; Mateo de Arias et al. 2020; Selva et al. 2020; Wyder et al. 2020; Fei et al. 2021), the most abundant Gene Ontology (GO) terms differentially expressed between sexual and apomictic genotypes belong to few categories. They are associated with stress response, and biotic and abiotic stimuli. Here we discuss the potential role of the relationships among the exogenous stresses and relative plant response in the sexual/apomictic determination.

## Oxidative stress

The oxidative stress hypothesis (Hörandl and Hadacek 2013) was proposed to explain the importance of sexual recombination. Reactive Oxygen Species (ROS) as the oxidative stress-related effectors activate meiosis-specific proteins that enhance the recombination frequency by causing double-strand breaks; meiotic sex could act as a cellular survival strategy (Hörandl and Hadacek 2013; Speijer et al. 2015).

In the facultative sexual green algae *Volvox carteri*, cellular ROS level increased twofold upon stress, regardless of the type of stress, causing the activation of sex genes and triggering sexual reproduction (Nedelcu et al. 2004). In general, GO terms related to “response to stress” are likely to be enriched in nucellar somatic embryony, typical of the genus *Citrus* (Kumar et al. 2014; Long et al. 2016). Activation of sex genes in response to stress has only been defined in polyembryonic ovules. Thus, oxidative stress may give cells the ability to induce nuclear embryogenesis and to express antioxidant enzymes such as superoxide and peroxidase (Long et al. 2016). In vitro, somatic embryogenesis can be triggered by oxidative stress derived from ROS (Ganesan and Jayabalan 2004; Cheng et al. 2015). A recent paper (Mateo de Arias et al. 2020) investigated the effect of oxidative stress on the transition between apomeiosis and meiosis. Drought, heat, starvation, and H_2_O_2_, all of which increase oxidative stress, also increase the rate of transitions from apomeiosis to meiosis. In contrast, the opposite was true when immature pistils were cultured on media including antioxidants, glucose, abscisic acid, fluridone, and 5-azacytidine that alleviate the ROS action. Apomeiotic spores and gametophyte formation (*Taraxacum*, *Antennaria,* and *Hieracium* types) was inducible in sexual *Arabidopsis thaliana*, *Antennaria dioica*, *Boechera gunnisoniana, B. stricta*, *B. exilis,* and *Vigna unguiculata* (Mateo de Arias et al. 2020)*.* ROS attenuation genes responsible for catalases (*CAT1*, *CAT3*), peroxidases (AT5G47000, etc.), and thioredoxins (*NTRA*, *CDSP32*, *QSOX2*), were upregulated in apomictic *Boechera spp. ANACO53* and *MMS ZWEI HOMOLOGUE2* (*MMZ2*), two genes that attenuate toxic conditions caused by ROS, were found upregulated in sexual *Boechera stricta,* confirming the view that meiosis evolved as a DNA repair mechanism (Mateo de Arias et al. 2020)*.* While some genes related to redox processes had higher expression levels in apomicts, others belonging to the same group such as the homolog of *AtPEROXIGENASE2* and another oxidoreductase (Bostr.7867s1594), were upregulated in sexual *Boechera* (Zühl et al. 2019). Interestingly, the cellular redox signaling hub has a multiple and robust connection to the hormones network (Considine and Foyer 2014; Klatt et al. 2016).

### Photoperiod

It has been shown that prolonged photoperiod, another biotic stress responsible for ROS production, increases the ratio between sexual and apomictic embryo sacs in various genera, i.e., *Themeda* (Evans and Knox 1969), *Dichanthium* (Saran and Dewet 1976), *Paspalum* (Quarin 1986) and *Ranunculus* (Klatt et al. 2016).

Klatt et al. (2016) investigated how a prolonged photoperiod could affect apomeiosis and parthenogenesis in *Ranunculus auricomus* clonal progenies. In their work, they analyzed the mode of reproduction after megasporogenesis and at the seed stage. They performed a targeted metabolomics analysis of phenolic metabolites with antioxidant functions, putatively linked to increased stress tolerance. Under 16.5 h of light, the plants showed different stress-related metabolite patterns, enriched in chlorophyll degradation products and various phenolic compounds. Plants also produced higher levels of sexually rather than asexually derived seeds (Klatt et al. 2016). These latter progenies showed considerable variation regarding megasporogenesis vs. apospory indicating epigenetics and transcriptional events as the main reasons for the phenotypic expression of apospory (Grimanelli 2012; Schmidt et al. 2014; Klatt et al. 2016). Ulum et al. (2020) further investigated the effect of extended photoperiod in diploid, tetraploid, and hexaploidy *Ranunculus auricomus* and showed how stress treatments significantly increased the level of sexual seeds in all cytotypes, with the most potent effect on diploids. This confirmed their hypothesis that megasporogenesis is modulated by light stress (more megaspores are formed compared to the control) and that polyploids better withstand environmental stresses, facilitating apomixis. It is worth noting that extended photoperiod affected apomeiosis but not parthenogenesis or endosperm development since there were no changes in the frequency of seed set nor proportion of sexually vs. asexually produced seeds (Ulum et al. 2020).

### Drought/heat/salt/osmotic stress

Mateo de Arias et al. (2020) tested the effects of drought and heat stresses on *Boechera lignifera* and *B. gunnisoniana.* Under these conditions, sexual tetrads in both taxa were observed two to threefold more frequently than diplosporous dyads. Shah et al. (2016) performed RNA-seq analysis on seedlings of *Boechera spp.* exposed to drought, salt, and osmotic stresses, finding that several genes were differentially expressed between the sexual and apomictic group. For example, they found that homologs of *ASYNAPTIC1* (*ASY1*) and *MULTIPOLAR SPINDLE 1* (*MPS1*), two critical meiotic genes, were downregulated in apomictic seedlings. ASY1 has a DNA binding domain involved in synapsis and crossover formation, and it is required for DMC1-mediated inter-homolog recombination during female and male meiosis (Sanchez-Moran et al. 2007). *MPS1* is essential for the spindle formation, and it is necessary for faithful chromosome segregation during female and male meiosis (Jiang et al. 2009). This was accompanied by the upregulation of stress-responsive genes belonging to the *LATE EMBRYOGENESIS ABUNDANT* (*LEA*) family and the *NAC-DOMAIN CONTAINING* (*NAC*) transcription family, which can regulate ovule separation in early reproductive development (Pedrosa et al. 2015). Among these, *NAC019* and *NAC055* seem to be expressed in floral buds and are ploidy independent. In addition, hormone pathways were differentially expressed in apomictic seedlings, with abscissic acid (ABA) and jasmonic acid (JA) biosynthesis and response genes upregulated while auxin and cytokinin downregulated. Taken together, these results suggest a better stress acclimation of apomictic over sexual seedlings, perhaps in a ploidy-dependent manner (Shah et al. 2016).

Preite et al. (2018) investigated the effects of drought and salicylic acid (SA) stresses on two apomictic dandelions (*Taraxacum spp.*) lineages: the former stress showed only a marginally significant effect (accession x drought effect) on methylation profiles and no transgenerational stability. Conversely, SA induced undirected methylation changes in offspring plants, possibly with a more complex mechanism than direct transmission from the mother plant. These results partially contrast with Verhoeven et al. (2010), whose results indicated that the environmental stress tested (salt, jasmonic acid, and salicylic acid) induce DNA methylation changes and can be faithfully transmitted to the offspring.

Under drought stress, two apomictic accessions of *Eragrostis curvula* showed a significant increase (from 2.4 and 4.0% to 14.4 and 22%, respectively) of sexual embryo sacs (Rodrigo et al. 2017). In the same species, Selva et al. (2020) found very similar percentages, and several differentially regulated genes. These included *ROS1A*, a DNA-glycosylase that demethylates cytosines, and the transcription factor *NAC10*, both previously related to apomixis.

Enhanced stress perception such as carbon starvation, increased salinity, and the suppression of ABA signaling establishes homeostasis in apomictic ovules. Still, this phenomenon could not be explained without other transcriptional shocks (Mateo de Arias et al. 2020). SnRK2.7 and SnRK3.22 are related to salt stress and were upregulated in apomictic *Boechera spp*. independently ABA and other Differentially Expressed genes (DEGs) when compared to sexually producing plants. (Mateo de Arias et al. 2020).

A phenotypic analysis recorded a difference in proline content during heat/drought stress and a two–threefold increase of apomictic plants in *Cenchrus* (Kumar et al. 2019), but epigenetics may not be involved. In the same species, in vitro salt stress seems to subdue apomixis through a higher proportion of sexually reduced embryo sacs (Gounaris et al. 1991).

In ferns, water scarcity, together with increased exposure to light, sugars, and hormones, seems to have shifted their reproductive mode toward apomixis in desert and monsoonal climates. At the same time, each of these factors alone was insufficient to induce apogamy (Grusz et al. 2021).

### Cold

As previously stated, some epigenetic modifications can cause more shifting from sexual to asexual reproduction. In *Ranunculus*, cold stress causes an increase in the number of unreduced gametes, a crucial step for apomixis and thus in polyploidization (Klatt et al. 2016; Schinkel et al. 2017). *R. kuepferi* is an aposporous species of *Ranunculaceae* with many cytotypes: diploid sexual populations in lower Alpine areas; tetraploid, usually apomictics, in the colder and higher niches of Alps and Apennines (Schinkel et al. 2020); triploids found only in the sympatric zones between the diploids and tetraploids (Cosendai and Hörandl 2010). Tetraploids have been molecularly dated to the last postglacial era (10–80,000 years ago), perfectly congruent with the high genetic homogeneity of these apomicts (Kirchheimer et al. 2018). Schinkel et al. (2020) found distinct epigenetic patterns related to cytotype and between the modes of reproduction in tetraploids. In other plant species, they found a correlation between methylation and an ecological gradient (altitude and annual mean temperature).

### Epigenetic phenomenon

We already introduced the importance of the epigenome machine in apomixis that is the link between the environment, the genes, and their asynchronous expression.

Epigenetic modifications, such as methylation, acetylation, and histone modifications, are constantly used by the cell to regulate transcription. In addition, several kinds of small RNAs operate against viruses and Transposable Elements (TEs), performing critical functions in development, stress responses, and transgenerational inheritance (Zhang et al. 2018).

In plants, epigenetic regulation can confer high phenotypic plasticity in response to environmental changes, a highly relevant topic due to global warming. Expression of epigenetic changes can be immediate, delayed within a generation, or expressed trans generationally via a sort of plant “memory” (Verhoeven and van Gurp 2012). The epigenome refers to modifications in the DNA structure without altering the sequence and can be inherited from parents through generations. Epigenetic modifications can be transmitted to subsequent generations (Johannes et al. 2009), although a reset, even if not complete, between generations often occurs, especially in absence of selection pressure (Tricker 2015; Anastasiadi et al. 2021; Ono and Kinoshita 2021). In apomictic plants, the reset may occur during gametogenesis and not in the early embryo development (Verhoeven and Preite 2014). The origin of epigenetic variation is stochastic and comparable to random genetic mutations subjected to natural selection (Shea et al. 2011; Verhoeven and Preite 2014) or induced by the environment (Dowen et al. 2012) in a targeted (non-random) way and may directly mediate plasticity (Becker et al. 2011). The heritability of DNA methylation is genotype-dependent and can last for at least two subsequent offspring generations (Preite et al. 2018). Targeted methylation appears less stable and more easily lost during generations than stochastic methylation (Hagmann et al. 2015; Preite et al. 2018). In both cases, heritable DNA methylation contributes to population differentiation along ecological gradients (Preite et al. 2015; Schinkel et al. 2020). Many of the known epialleles are associated with the silencing of TEs that can affect the expression of nearby genes (Paszkowski and Grossniklaus 2011; Preite et al. 2015).

However, disentangling epigenetics from genetic variation can be a challenging task that requires specific experimental schemes and apposite populations (Johannes et al. 2008; Verhoeven et al. 2010); this can be better studied and understood in populations with no genetic variations, i.e., clonal populations (Johannes et al. 2009). Some asexual lineages can be very successful in adapting to specific conditions or new habitats and this is believed to be attributed to epigenetic changes (Verhoeven and van Gurp 2012; Richards et al. 2012; Verhoeven and Preite 2014).

Sun et al. (2004) showed that, in *Arabidopsis*, the female germline tissues withstood environmental stresses better than male tissues. This supports the idea that maternal sporophytic tissues can lead to a genetic/epigenetic shift and to a switch between sexual and apomictic reproduction, upon exposure to certain stresses (Shah et al. 2016). For example, in *Boechera,* several ovules failed to produce seeds under stress conditions, while almost all seeds were formed apomictically (Mateo de Arias et al. 2020). Faster growth of apomictic derived embryos reduced sexual progenies in aposporous plants because of competition between embryo types within the same ovule (Hojsgaard et al. 2014). This effect was much stronger in diplosporous plants because the archespore cell chooses only one developmental fate (Hojsgaard et al. 2014; Rodrigo et al. 2017). Due to this, one can expect that facultative apomictics will switch to sexual reproduction under a stress condition to facilitate adaptation (Rodrigo et al. 2017). Adversely, the reproduction of facultative asexual plants is often determined by genotype x environment interactions (Hörandl and Hadacek 2013). The reprogramming occurs within a few hours of stress induction with meiosis I being the most sensitive to oxidative stress and most likely the stage for shifting apomeiosis to meiosis (Mateo de Arias et al. 2020). At the same time, it is possible that environmental conditions can change during the life cycle of the plant (Karunarathne et al. 2020). Variables such as mean diurnal range, annual temperature range, and precipitation can be correlated with differing proportions of apomictic and sexual pathways (Karunarathne et al. 2020), thus introducing a type of seasonal variation in reproduction patterns.

### Small RNAs

Small RNAs (sRNAs) are a class of endogenous single-stranded, non-coding RNAs, typically 20–24 nucleotides long, produced by genes distinct from the ones that they regulate (Amiteye et al. 2011). Among them, miRNAs are the most studied, and are known to down-regulate the expression of target genes at the post-transcriptional level via translational inhibition or mRNA cleavage (Voinnet 2009). Many miRNA families act on critical biological processes, e.g., signaling and hormone pathways, stress response, and plant and flower development (Galla et al. 2013). Small RNAs pathways are involved in both sexual and apomictic developmental programs as proposed by Olmedo-Monfil et al. (2010), Amiteye et al. (2011), Singh et al. (2011), Tucker et al. (2012), Rabiger et al. (2016), Selva et al. (2017), and Long et al. (2019), and can contribute to sporophytic apomixis (Long et al. 2016). For example, the expression of miR172 changes during the developmental stages of sporophytic apomictic *Zanthoxylum bungeanum* fruits 15 days after flowering starts by inhibiting the activity of *TOE3* and *AP2* and increasing the expression of *AGAMOUS* in S3 corresponding to young fruit (Fei et al. 2021). Small RNAs and mRNAs can move from somatic to ovule cells (Tucker et al. 2012), or from the roots to the vegetative tissues, and influence various processes (Thieme et al. 2015). To investigate the potential role of long-distance signaling on apomixis, Rabiger et al. (2016) grafted apomictic plants on sexual plants, and vice versa, demonstrating that apomixis is not transmissible by grafting. This suggests that signals required to induce the development of the Aposporous Initial Cells (AICs) are more likely to come from floral and ovary tissues than long-distance signaling.

### Methylation

The transcriptional reprogramming associated with RNA-dependent DNA methylation (RdDM) pathways appears to be a crucial step in acquiring and maintaining apomictic reproduction (Verhoeven et al. 2010; Hand and Koltunow 2014; Podio et al. 2014). Sexual development could rely on the recognition between the sRNAs and their targets. For example, in hybrids, sRNA sequences tend to diverge, resulting in a lack of targeting of TEs in undifferentiated cells (Armenta-Medina et al. 2011). Consistently, selective activation of TEs between sexual and apomictic genotypes has been found by Garbus et al. (2017). Members of the *ARGONAUTE4* clade in *Arabidopsis thaliana* encode proteins binding heterochromatic small interfering RNAs (siRNAs) that target repeat sequences and transposable elements (Selva et al. 2017). The loss of function mutant *ago9* resulted in ovules with multiple archespores that short-circuit sporogenesis (Olmedo-Monfil et al. 2010), while *HpAGO9* expression is significantly reduced in *Hypericum perforatum* aposporic pistils (Galla et al. 2015, 2017). This suggests that a trans-acting small interfering RNA (ta-siRNA) pathway can influence the female gametophytic lineage and simulate apospory. This is the case of *TEX1* and TAS3 that restrict the expression of *ARF3* through the *TAS3*-derived ta-siRNA: when a mutation in the THO*/*TREX complex occur, *ARF3* expression is not limited to the medio chalaza domain of ovule primordia but also to epidermal cells, resulting in the formation of multiple megaspore mother cells (Su et al. 2017). Conversely, ZmAGO104 is necessary for non-CG methylation of centromeric and knob-repeat DNA (Singh et al. 2011) and the loss of function *ago104* maize mutant produces up to 70% of functional unreduced gametes resembling diplospory. Simultaneously, a semi-dominant mutant of *ARGONAUTE5* (*AGO5*), a putative effector of the sRNA silencing pathways, showed defects in the initiation of megagametogenesis in sexual plants (Tucker et al. 2012). Finally, a mutation in the *AGO5* orthologue *MEL1* of maize causes the arrest of meiosis and male sterility (Komiya et al. 2014).

Deregulation of *ZmCHR106*, homolog of *AtDDM1* (*DECREASE IN DNA METHYLATION*), led to an apomixis-like phenotype (Garcia-Aguilar et al. 2010). *ZmDDM1* is critical for DNA methylation at CHG sites, and to a lesser extent, at CG sites, in heterochromatic regions and it is also required to form ^m^CHH islands. Furthermore, *ZmDDM1* is necessary for the presence of 24-nt siRNA, suggesting its involvement in the RdDM pathway (Long et al. 2019).

Selva et al. (2017) explored floral transcriptomes of sexual and apomictic *Eragrostis curvula* to identify homologs of these genes and compare their spatial and temporal expression. They found that homologs of *AtAGO9*/*ZmAGO104*, *AtCMT3*/*ZmDMT102*/*ZmDMT105A*, and *AtDDM1*/*ZmCHR106* genes, showed contrasting expression patterns between apomictic and sexual individuals. Because of this, they hypothesized that the ectopic expression of *EcAGO104* in *Eragrostis curvula* archespores could promote gametophytic development, avoiding meiosis. Their results suggest that in *Eragrostis curvula,* altered localization of *AtAGO9*/*ZmAGO104* expression is more important than an RdDM breakdown in the ovule to achieve diplospory. Using in situ hybridization, a signal was detected for *EcDMT102* in ovules representing a possible antisense regulation through a sense-antisense mRNA complex (Selva et al. 2017) previously observed in apomictic plants with *EMBRYOGENESIS RECEPTOR KINASE* (Podio et al. 2014) and *ORIGIN RECOGNITION COMPLEX3* (Siena et al. 2016). Moreover, eight *AGO* transcripts were expressed explicitly in apomictic *Boehmeria tricuspis,* especially in the AII phase (Tang et al. 2017).

*CMT3* expression of *Arabidopsis* is crucial for egg-specific silencing of eu/heterochromatic domains. Indeed, *cmt3* mutant results in a phenotype similar to *ago9,* with heterochromatic transcription in the egg and the reactivation of transposable elements (Pillot et al. 2010). *CMT3* expression decreased during pistil development in apomictic *Hypericum* but not in its sexual counterpart (Galla et al. 2017). In the same plant system, *MET1* appeared to be differentially expressed in apomictic and sexual pistils during gametogenesis (Galla et al. 2015), with the loss of function of *AtMET1* leading to the removal of silencing methylation marks and enhanced expression of genes, such as *FIS2* and *FWA*, involved in the regulation of endosperm development. In other words, *MET1* inhibits endosperm development in the absence of fertilization in *Arabidopsis* (Schmidt et al. 2013).

Other genes whose deregulation caused a phenotype resembling apomixis are *RNA-DEPENDENT RNA POLYMERASE2* (*RDR2*), *DICER*-*LIKE3* and *4* (*DCL3* and *DCL4*), and *DNA*-*DIRECTED RNA POLYMERASE* V and VI (POL V and POL VI) of *Arabidopsis*. These genes are all involved in regulating 24-nt siRNAs biogenesis, as part of the RdDM pathway (Rabiger et al. 2016; Selva et al. 2017). Mutations in *RDR6* and *SUPPRESSOR OF GENE SILENCING3* (*SGS3*) induce the formation of extra gametic precursor cells, as both genes are required to produce 21-nt siRNAs and ta-siRNAs (Olmedo-Monfil et al. 2010; Rabiger et al. 2016). To better understand the interactions between these genes and their control of cell specification, we refer to Kawashima and Berger (2014) and Wang and Kohler (2017).

In *Boechera spp*, RdDM silencing and polycomb repressive complex activity, were upregulated in sexual ovules compared to apomictic ones (Mateo de Arias et al. 2020). *IDN2* (*INVOLVED IN *DE NOVO* 2*) is a double-stranded RNA-binding protein that binds to POL V-produced non-coding RNAs. *IND2* is involved in de novo methylation and siRNA-mediated maintenance of methylation and was downregulated in apomictic pistils compared to sexual ones (Galla et al. 2017). Features of apospory have been observed in *Arabidopsis* mutants for the RNA helicase *MNEME* (*MEM*), which permits only one germinal cell per ovule (Schmidt et al. 2011, 2014).

In-depth characterization of the *Paspalum simplex* Apomixis Controlling Locus revealed the presence of repetitive elements, gene degeneration (Calderini et al. 2006), and deregulation (Polegri et al. 2010), with a high level of methylation (Podio et al. 2014). An essential role of methylation in the control of parthenogenesis was proven in *Boechera stricta*, *Arabidopsis,* and cowpea by Mateo de Arias et al. (2020). In both papers, treatment with the demethylating agent 5’-azaC enhanced the frequency of dyads and diplosporous embryo sac.

### Transposons and retrotransposons

Several transposons and retrotransposons are speculated to be involved in the apomictic process. For example, in *Paspalum notatum* Laspina et al. (2008) found that several genes belonging to transposon activity (a transposon protein and a putative transposase) were expressed in sexual but not in apomictic genotypes and vice versa for retrotransposon proteins. In *Eragrostis curvula,* a MADS-box transcription factor and a transposon were specifically repressed in sexual genotypes, likely due to interactions with miRNAs (Garbus et al. 2019). Retrotransposon activity, in particular, should be regarded as a consequence of recombination suppression around apomixis loci (Hojsgaard 2020). The retrotransposon family Opie-2-like is particularly abundant within the ASGR of *Pennisetum squamulatum* (Akiyama et al. 2004) and in all the chromosomes of *Cenchrus ciliaris* (Akiyama et al. 2005)*.* In the same species*,* six retrotransposons showed apomixis-associated activity (Kumar 2017) and a Gy163 transposon was hypomethylated and thus overexpressed in apomictic vs. sexual reproductive tissues (Rathore et al. 2020). The authors hypothesized that demethylation in the coding region of Gy163 could lead to apomictic seed development.

### Histone modifications and variants

Plant epigenomics rely on many different mechanisms that are related to one another. Different histone modifications can alter the “histone code”, promoting either active transcription or gene silencing. For example, manipulation of *CENH3* can induce genome elimination and it is possible that *CENH3* levels vary naturally in apomictic systems. Differing expression levels of *CENH3* were found in sexual *Arabidopsis* when compared to apomictic *Boechera* (Schmidt et al. 2014). The authors hypothesized that the absence of *DYAD*/*SWITCH* together with high expression levels of *CENH3* could explain naturally occurring diplospory. *CENH3* could also hinder the paternal contribution to the offspring (Schmidt et al. 2014). Additional differentially expressed epigenetic regulators have been found in maize-*Tripsacum* hybrids: *SWI2*/*SNF2*-like chromatin remodeler *CHR106*, histone H1 linker *HON101*; histone deacetylase *HDT104*, and three DNA methyltransferases, *DMT102*, *DMT103* and *DMT105* (Garcia-Aguilar et al. 2010). *DMT*102 and *DMT103* are expressed in germ cells and the surrounding nucellar cells, and knock-out mutants for these genes can form extra embryo sacs and/or unreduced gametes and show a localized release of a repressive chromatin state. The chromatin state in *dmt102* mutant is associated with *H3K9* hyperacetylation, an epigenetic signature that has also been found in ovules of apomictic plants (Garcia-Aguilar et al. 2010).

### Endogenous factors/effectors

#### Hormones

Auxin biosynthesis is necessary for proper growth of the female gametophyte (Panoli et al. 2015). Additionally, auxin is differentially regulated in sexual and apomictic plants (Yamada-Akiyama et al. 2009; Polegri et al. 2010; Koltunow et al. 2011; Schmidt et al. 2014; Galla et al. 2017; Ortiz et al. 2017, 2019; Mateo de Arias et al. 2020). Data suggest that altered regulation of the auxin biosynthetic pathway is likely concomitant with aposporous gametophyte development (Koltunow et al. 2011; Okada et al. 2013; Galla et al. 2017). According to this hypothesis, *BbrizSec13* and *BbrizRan*, two sequences from *Brachiaria brizantha* ovaries, may be related to specification of the nucellar cells that will form the megagametophyte of apomictics through an auxin pathway (Silveira et al. 2012). In sexual *Arabidopsis*, *SPOROCYTELESS* (*SPL*) represses the expression of two *YUCCA* genes that encode for proteins crucial for auxin biosynthesis (Li et al. 2008), and *spl* mutants showed altered differentiation of the sporogenic primary cells and an altered gametophytic phase (Yang et al. 1999). The previously cited *AGO9* also interacts with the miR390 and miR167 families, both regulating auxin responses (Armenta-Medina et al. 2011). miRNAs regulate transcription factors that interact with many hormones (e.g., *WRKY75*) or directly in the auxin regulatory pathway (*AVP1*, Fei et al. 2021).

In apomictic *Paspalum notatum,* Ortiz et al. (2019) found several genes associated with plant reproductive development, auxin/cytokinin signaling, transcription control, and biomolecules transport differentially epi-regulated between sexual and apomictic genotypes. The authors suggest further investigating the role of miR160, miR167, and miR319 families that were also found differentially expressed. Moreover, in a different study they identified 14 genes involved in auxin transport and metabolic pathways with altered expression between sexual and apomictic individuals, reinforcing what they previously proposed for the role of auxin as a key player in the establishment of apomixis (Ortiz et al. 2017). The importance of the auxin signaling pathway has also been highlighted by Schimdt et al. (2014) as genes involved in auxin signaling, belonging to AUX/IAA and ARF transcription factor families, were found upregulated in apomictic *Boechera* genotypes. *ARF* genes were also found differentially regulated in *Paspalum simplex* (Polegri et al. 2010). In addition, *ARF2*, *ARF7*, together with *TRN2*, *CPL2*, and *ASK2* that are involved in the regulation of auxin homeostasis, were significantly downregulated in apomictic vs. sexual *Hypericum perforatum* where *IAA4* and *SAUR20* were, instead, upregulated (Galla et al. 2017). In *Cenchrus ciliaris,* the parthenogenetic eggs may differ from the sexual ones in auxin perception as several genes for auxin receptors and negative auxin regulators were found upregulated in parthenogenetic eggs (Ke et al. 2021). *ETHYLENE INSENSITIVE 2* and 14 ethylene-responsive factors were also found to be upregulated in parthenogenetic vs. sexual eggs (Ke et al. 2021). In ferns, ethylene is likely to regulate apogamous sporophyte development, but only with sufficient light and sucrose to provide the energy for activities directed by ethylene (Elmore and Whittier 1975).

Several authors have speculated that the shift from regular meiosis into apomeiosis may involve auxin and stress response genes. For example, *SERK* (*SOMATIC EMBRYOGENESIS RECEPTOR-LIKE KINASE*) genes are believed to participate in the specification of Aposporic Initial Cells (Albertini et al. 2005; Podio et al. 2014). Moreover, *SERK3* and *BRI1-ASSOCIATED RECEPTOR KINASE* (*BAK1*), which enhance brassinosteroids (BR) transduction signaling, were found upregulated in sexual *Boechera stricta*. Brassinosteroids could play a role in apomictic development as they can be involved in growth and expansion of the AICs (Galla et al. 2017; Rabiger et al. 2016) or with megagametogenesis (Gruszka 2020). Several genes involved in BR biosynthesis, i.e., *CYP85A2* and *ELL1*, were found differentially expressed in sexual vs. apomictic *Boechera* spp. by Mateo de Arias et al. (2015, 2020). Recently Mateo de Arias et al. (2020) found 85 enriched GO categories related to BR upregulated in apomictic *Boechera* plants. In facultative *Eragrostis curvula,* Selva et al. (2020) found that *EXORDIUM* and two other genes of the brassinosteroids pathway, were differentially expressed in drought-stressed plants showing an enhanced level of sexual reproduction.

Another interesting hormone related to apomixis is Abscisic acid (ABA). This hormone is closely related to ROS synthesis, signaling, and other genes involved in the antioxidants network and could prevent programmed cell death. Ferreira de Carvalho et al. (2016) speculated on its involvement in pursuing an ancestral cell fate converging into gametophyte formation. Apomictic plants are likely to chronically upregulate genes of the ABA pathway, which in turn upregulate stress attenuation processes. It has been hypothesized that the level of stress attenuation in ovules causes a sex/apomixis switch in angiosperms, with high levels of stress attenuation suppressing meiosis and inducing apomeiosis, and low levels of stress attenuation having the opposite effects (Mateo de Arias et al. 2020). Stress attenuation conditions that revert meiosis into apomixis seem to boost expression of genes involved in biosynthesis of ABA itself (e.g., *ABA DEFICIENT2* and *DIVARICATA2*) or decrease those involved in ABA catabolism (*ABA INSENSITIVE 4*, Mateo de Arias et al. 2020). ABA receptors, such as *ABA-INDUCED TRANSCRIPTION REPRESSOR 2*, were also found upregulated in apomictic *Boechera formosa* (Mateo de Arias et al. 2020). In *Boechera*, Zühl et al. (2019) found that the *HISTIDINE KINASE 1* (a gene of the ABA pathway involved in drought stress response), and GH3 (an auxin-responsive family protein) were differentially expressed between apomictic and sexual plants. Sucrose non-fermenting-1 (*SNF1*)-related kinase 1 (*SnRK1*) is a stress response gene. It is part of a signaling network that comprises the ABA and TOR (TARGET OF RAPAMYCIN) kinase pathways, and *SnEK1* is differentially expressed in apomictic and sexual *Eragrostis curvula* under drought conditions (Selva et al. 2020). TOR stimulates the production of BR and is a regulator of bioenergetics. It can be inhibited by ROS and its inactivation suppresses growth (Mateo de Arias et al. 2020).

Contrary to auxins, cytokinins negatively regulate cell proliferation in the sporophytic tissues surrounding the developing embryo sac (Cheng et al. 2013), and their degradation is likely to be increased in apomictics (Schmidt et al. 2014; Galla et al. 2017; Ortiz et al. 2019). Galla et al. (2017) found several cytokinin genes, such as *HPCHC1*, *IPT2*, and *ACR4*, downregulated in apomictic *Hypericum perforatum* genotypes compared to sexual genotypes. The two former genes are associated with cytokinin catabolism, while the latter is associated with cytokinin signaling. Genes involved in cytokinin degradation were found to be upregulated in apomictic *Boechera* plants compared to their sexual counterpart by Schmidt et al. (2014).

Gibberellins were also linked to apomixis. For example, the *GID1* (*GIBBERELLIN-INSENSITIVE DWARF1*) gene is a gibberellin receptor that is expressed in the nucellus of apomictic *Brachiaria brizantha* before AICs differentiation (Ferreira et al. 2018). During *Paspalum notatum* anthesis, *GID1* showed an increased expression in apomictic plants, while it was expressed at low levels in sexual ones (Podio et al. 2021). In *Arabidopsis*, *GID1a* overexpression triggers the differentiation of MMC-like cells, suggesting its involvement in ovule development (Ferreira et al. 2018).

Jasmonic acid pathway genes are also upregulated in apomictics (Okada et al. 2013; Galla et al. 2017). As of now the previously cited hormone pathways, including ethylene and salicylic acid, are indicated by Tang et al. (2017) as specific regulators of diplospory response.

Ca2 + and calmodulin are not hormones, but act as second messengers in a variety of processes (Thor 2019) among which the fertilization and the egg activation. Genes related to calcium were found exclusively upregulated in parthenogenetic eggs compared to sexual eggs of *Cenchrus ciliaris*, suggesting a calcium-triggered pathway in parthenogenesis (Ke et al. 2021).

### Polyamines

In *Arabidopsis*, spermidine synthesis is crucial for embryo development (Imai et al. 2004), and it also seems to be involved in somatic embryogenesis (Schmidt et al. 2014). These compounds are involved in responses to several stresses and diseases (Chen et al. 2019). According to Schimdt et al. (2014) spermidine accumulation can strengthen apomictic plants against oxidative stresses. Genes responsible for the biosynthesis of spermidine and other polyamines were highly expressed in the apomictic gametophyte with respect to sexual ones (Schmidt et al. 2014). LOC_Os04g31000.1, a putative gene for spermidine synthase has been identified in *Pennisetum squamulatum* ASGR (Conner et al. 2008).

### Carbohydrates and lipids

Carbohydrate and lipid metabolism genes were found differentially regulated in *Hypericum perforatum* by Galla et al. (2015, 2017) and in *Boehemeria tricuspis* by Tang et al. (2017). Likewise, AICs of *Boechera gunnisoniana* are enriched in terms that are ontologically associated with carbohydrate transport, membrane lipids biosynthesis, biosynthesis, and transport of amino acids (Schmidt et al. 2014). The sugar transporter *ZIF2*, also called *ERD6*, was found differentially expressed between sexual and apomictic *Boechera by* Mateo de Arias (2015). Moreover, genes codifying for the catabolism of amino and fatty acids as well as carbohydrates have been found upregulated in apomictic *Boechera* (Mateo de Arias et al. 2020).

### Proteins ubiquitination

Ubiquitination regulates nearly all of the cellular cycle, allocating intracellular proteins to activity adjustment or proteolysis. Protein turnover has been demonstrated to influence reproductive fate and germline development (Thomann et al. 2005). Ubiquitin-related genes have been cited as involved in apomixis in *Paspalum notatum* (Laspina et al. 2008), *E. curvula* (Cervigni et al. 2008; Garbus et al. 2017), and *Boehmeria tricuspis* (Tang et al. 2017). *ARI* family members such as *ARI7*, involved in ubiquitin-dependent protein degradation, showed higher activity in sexual *Arabidopsis thaliana* MMCs compared to *Boechera gunnisoniana* AICs (Schmidt et al. 2014).

Regarding protein turnover, many genes were also differentially regulated in *Paspalum notatum*, with the ribosomal protein S12 and the ribosomal protein L35-like both upregulated in the apomictic plants (Laspina et al. 2008). These results are in contrast with those found by Albertini et al. (2004), who found S12 expressed only in sexual *Poa pratensis*.

Protein degradation pathway genes, codifying for F-box proteins, E3 ligases-like, the homolog of *RMA3* and a TRAF-like superfamily protein, and ubiquitin-like superfamily protein like *UBC28*, were found to be upregulated in apomictic nucella of *Boechera* (Zühl et al. 2019). The ubiquitination pathway was differentially expressed in *Eragrostis curvula* (Carballo et al. 2021) under drought stress, with five genes down- and two upregulated (Selva et al. 2020). The Apospory locus (*HAPPY*) of *Hypericum perforatum* includes a homolog of *ARIADNE7*, a protein involved in regulatory processes and protein degradation (Schallau et al. 2010).

Bocchini et al. (2018) found that the hypo-methylation of PN_SCD1, a vesicle trafficking regulator orthologue of *Arabidopsis SCD1* (*STOMATAL CYTOKINESIS-DEFECTIVE1*, (Falbel et al. 2003), resulted in its upregulation in the nucellus of *Paspalum* apomictic plants just before the onset of apospory. They also found that several *PN_SCD1* biological partners (*RAB* proteins, *MAP3Ks*, clathrin and *CUL4*-based E3 ubiquitin ligases) were upregulated in apomictic genotypes. The vesicle trafficking is active in both apomictic and sexual plants but apomixis operates on the pathway directing protein localization in particular ways.

Overall, the ubiquitination pathway and its methylation can regulate the abundance of plant proteins, their activity, and subcellular location with a cascade that is a plausible explanation for a complex trait such as apomixis.

### Cell walls modification

Physical and molecular isolation through callose deposition allows a somatic cell to reprogram its fate (Parra-Vega et al. 2015) and callose deposition has been shown to be promoted by ROS (Luna et al. 2011). In *Boechera gunnisoniana*, genes involved in cell wall modification were found enriched in AICs with respect to the surrounding sporophytic nucellus tissues (Schmidt et al. 2014). Four genes responsible for cell wall modifications, *UGT*, *LAC*, ACHI, and *CHI*, were also upregulated in polyembryonic ovules of *Citrus* when compared with monoembryonic ovules (Long et al. 2016). In particular, *LAC* and *UGT* may contribute to the isolation of nucellar embryony initial cells, as the former encodes for a laccase necessary for lignin polymerization and *UGT* may be involved in callose biosynthesis. Similarly, in *Paspalum notatum,* Laspina et al. (2008) recorded differential expression of genes related to cell walls, such as Os03g52150, a significant surface like glycoprotein. Under drought stress, many genes involved in cell wall modifications and signal transduction were upregulated in sexual and downregulated in apomictic *Eragrostis curvula* (Selva et al. 2020). Moreover, genes involved in the cutin, suberin, and wax pathways were enriched in several comparisons between different stages of apomictic and sexual *Boehmeria tricuspis* (Tang et al. 2017). *EXO*-like genes required for cell growth and brassinosteroid-induced signaling appear to be secreted into the cell wall. The upregulation of *HpEXO*-like genes has been detected in aposporous *H. pilosella*, where it may promote the growth and expansion of the AI cell (Rabiger et al. 2016). The role of *HpLOX2*, a putative *LIPOXYGENASE* (*LOX*)-like gene, which was previously stated as associated with the AI cell (Okada et al. 2013), was confirmed by Rabiger et al. (2016).

## Conclusions

**Fig. 1:**
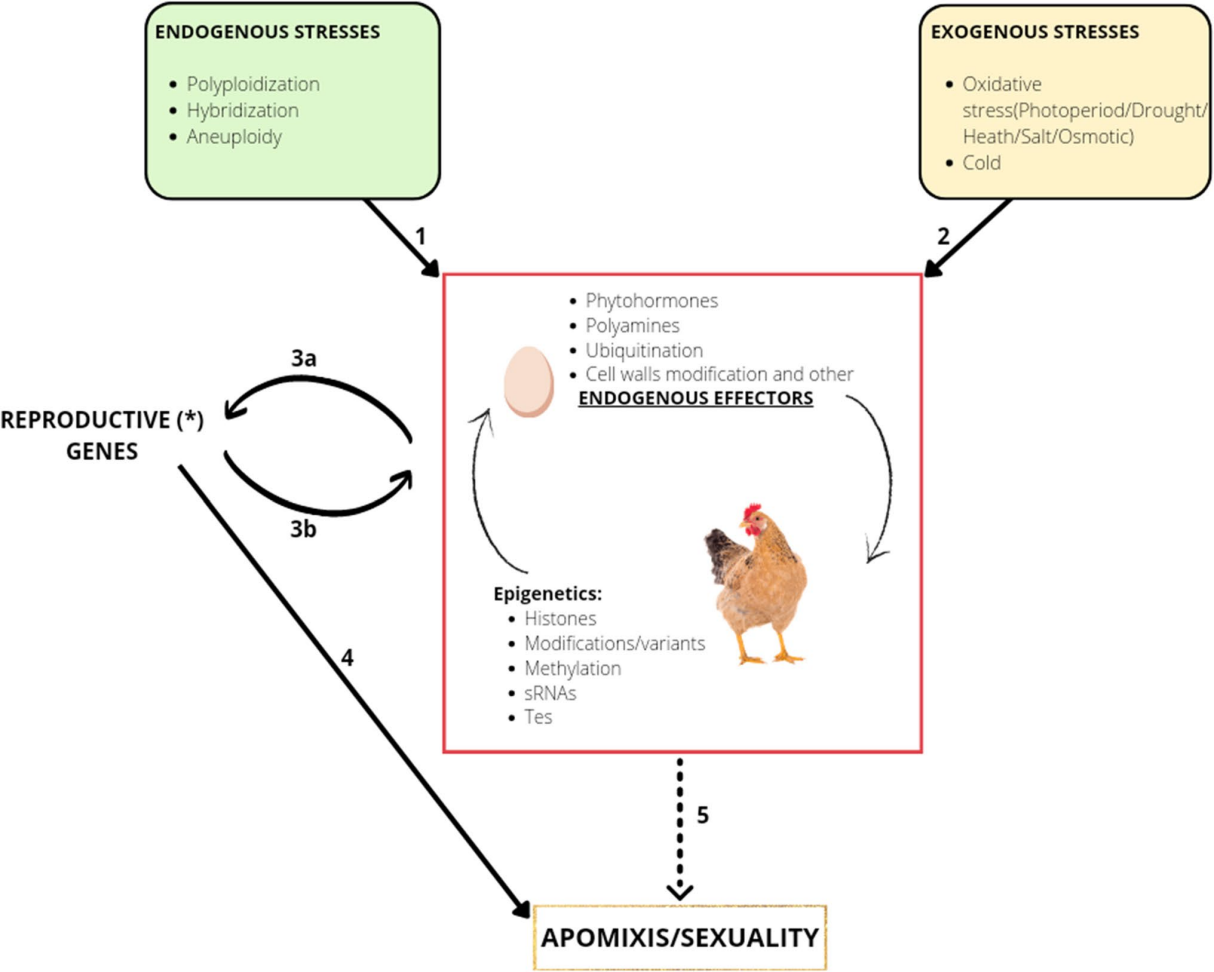
A hypothetical scheme for the regulation of apomixis. Endogenous and exogenous stresses can activate the epigenetic machine and/or the endogenous effectors such the phytohormones, the ubiquitination or the cell walls modification. (*) According to Mateo de Arias et al. (2020), reproductive genes could be replaced by metabolic homeostasis genes

All in all, phytohormones (with other endogenous effectors) and epigenetic regulation can interact on multiple levels through a crosstalk mechanism. The phytohormone signaling directly affects expression or activity of important chromatin modifiers and vice versa as chromatin machinery targets genes of the phytohormone metabolic/signaling pathways. At the same time, both players interact with genes involved in developmental or stress responses, resulting in the “chicken-and-egg” situation (Maury et al. 2019) illustrated in Fig. 1.

The complexity in the regulation and the fine-tuning of apomixis is extensive, where endogenous regulations can be seen as the central part of the event cascade leading to the establishment of apomixis. Cross-talking with chromatin regulation, ROS, and hormone signaling is central at a cellular level, as most stress response pathways converge into the H_2_O_2_ signaling pathway (Kovtun et al. 2000).

Many candidate genes have already been discussed in the introduction and the genetic hypothesis (Fig. 1 arrows 3b and 4) and are surely involved in the settlement of apomixis, though still not sufficient to explain the entire apomictic phenomenon. We also saw how endogenous stresses such as polyploidization, hybridization, and aneuploidy create shifts in time and space. This cause dosage-dependent disparities that are closely linked to miRNAs and are the translation of the Asynchronous hypothesis (arrow 1). The starting signal can come from miRNAs, but also from environmental, and hence exogenous, stresses (arrow 2). In both cases, it reflects downstream on phytohormones and epigenetic regulation. This complex can interact with genes related to meiosis and syngamy as well as metabolic homeostasis, they can then regulate the reproductive determination (3a), or vice versa genes can play a role in feedback regulation on the complex itself (3b). Mateo de Arias et al. (2020) tried to combine the Asynchronous and Polyphenic hypotheses by replacing the asynchronies in germline development with dosage-dependent disparities in metabolic homeostasis. The authors hypothesized that imbalances in gene expression resulting from hybridization, polyploidization, or other chromosome aberrations could induce a permanent stress-like state of metabolic homeostasis in ovules of apomictics (Mateo de Arias et al. 2020).

Enhancing a plant's ability to attenuate exogenous oxidative stresses could boost the capability of various plants (*Boechera*, *Vigna*, *Arabidopsis*; Mateo de Arias et al. 2020) to shift from sexual to apomictic meiosis depending on the capacities of the species in question. Without mutation in sexual *Boechera spp.,* the decrease in oxidative stress leads to apomixis, while increased oxidative stress shifts apomictic phenotypes to sexuality. In other words, the shifts from meiosis to apomeiosis could be reached only by changing metabolic conditions (Mateo de Arias et al. 2020). However, it is difficult to reproduce the state of stress attenuation typical of apomictics in sexual plants because the conditions are not induced by environmental factors. Genomic aberrations such as polyploidization, recombination or hybridization can lead to different levels of stress attenuation and hence to the broad range of facultative to obligate apomixis (Mateo de Arias et al. 2020).

Stress conditions, oxidative stress in particular, (and their attenuation) are likely to be involved in reproductive decisions as they influence the metabolic status of ovules and the genes that affect this status. Metabolic fluctuations may regulate the developmental sex/apomixis decision: in this hypothetical landscape, we can conjecture that the type of apomixis expressed is a function of the temporal and spatial expression, endorsing both the asynchronous (Hojsgaard 2020) and polyphenic hypothesis (Albertini et al. 2019). For example, *Boechera* nucellar cells can have four different developmental fates that we currently know of: meiosis, apospory, diplospory, and apoptosis, depending on the oxidative status of the pistil (Mateo de Arias et al. 2020). The pistil culture procedures used by Mateo de Arias et al. (2020) could not be used to test whether unreduced embryo sacs produced by environmental stress attenuation alone, because they were insufficient to obtain viable embryos and endosperm (Mateo de Arias 2015; Mateo de Arias et al. 2020). Coupling this kind of stress attenuation with mutations that enhance parthenogenesis and the autonomous development of the endosperm, we could obtain a useful genotype with full penetrance. Finally, the dashed arrow (Fig. 1) indicates a possible bypass of the genetic hypothesis if we interpret the genes related only to meiosis.

Conversely, if we also refer to homeostasis regulation genes, the three hypotheses converge, and there is no need for arrow 5. There is no evidence that the three theories proposed here contradict one another; on the contrary, combining them seems to explain all clues gathered to date. In this framework, combining candidate genes *stricto *sensu with genes that regulate metabolic homeostasis (the ovules of apomictic plants can detoxify the oxidative stress and induce apomixis) and/or genomic shocks could be a powerful strategy to achieve the complete penetrance of apomixis. More than 120 Gene Ontology terms related to carbohydrate, fatty acid, and protein metabolism processes, regulation of ROS, and other stresses were upregulated in apomictic *Boechera lignifera,* with respect to its sexual counterpart (Mateo de Arias et al. 2020). The number of genes involved is massive. Still, a first approach could come from a comparison of different datasets (e.g., Podio et al. 2021) by selecting among the upregulated genes in several apomictic vs. sexual plants, then overexpressing them with a strong promoter could help to understand their role in apomixis. In any case, such genetic engineering modifications should address metabolic homeostasis at a cellular level throughout ovule and early seed development to boost apomeiosis and parthenogenesis penetrance.

Finally, to further complicate an already intricate picture, a link between stress response and meiosis much earlier than the onset of reproduction itself has been supposed, as indicated by the fluctuations of the expression levels of *ASY1*, *MPS1* and *DYAD* during the vegetative phase (Shah et al. 2016). A precocious formation of embryo sacs and meiosis is likely typical of apomictic genotypes (Carman et al. 2011). Could investigating the epigenomics and the transcriptome of apomictic and sexual species from the early plant development onward be a solution to outline a model? We believe new holistic approaches capable of considering many covariates are needed to analyze apomixis mechanisms and its origin further.

### *Author contribution statement*

Conceptualization NT, EA Literature search NT Writing—original draft preparation NT Writing—review and editing NT, AA, EA. All authors gave final approval for publication and agreed to be held accountable for the work performed therein.

## Data Availability

Data sharing not applicable to this article as no datasets were generated or analyzed during the current study.
