# Correction to: Apomixis: oh, what a tangled web we have!

**DOI:** 10.1007/s00425-023-04130-2

**Published:** 2023-04-19

**Authors:** Niccolò Terzaroli, Aaron W. Anderson, Emidio Albertini

**Affiliations:** 1 Dipartimento di Scienze Agrarie, Alimentari e Ambientali, Università degli Studi di Perugia, Borgo XX Giugno 74, 06121 Perugia, Italy; 2 Fulbright Scholar From Department of Plant Sciences, University of California, Davis, USA; 3 Consorzio Interuniversitario per le Biotecnologie (CIB), Trieste, Italy

**Correction to: Planta (2023) 257:92** 10.1007/s00425-023-04124-0

In the original version of this article, the given and family names of [Terzaroli Niccolò and Albertini Emidio] were incorrectly structured. The correct name should read as Niccolò Terzaroli and Emidio Albertini.

